# A Highly Efficient White Luminescent Zinc (II) Based Metallopolymer by RGB Approach

**DOI:** 10.3390/polym11101712

**Published:** 2019-10-18

**Authors:** Barbara Panunzi, Rosita Diana, Ugo Caruso

**Affiliations:** 1 Department of Agriculture, University of Napoli Federico II, 80055 Portici NA, Italy; barbara.panunzi@unina.it (B.P.); rosita.diana@unina.it (R.D.); 2 Department of Chemical Sciences, University of Napoli Federico II, 80126 Napoli, Italy

**Keywords:** RGB, Zinc metallopolymer, PLQYs, LECs, WPLEDs

## Abstract

Three aryl-hydrazone *O*,*N*,*O* tridentate ligands with a different electron-withdrawing substituent were prepared. The introduction of a flexible charged chain in the ligands guaranteed solubility in many organic solvents and in water. The increasing withdrawing aptitude of the substituents red-shifted the emission in the correspondent metallopolymers. The metallated polymers were obtained by grafting ligand-zinc (II) coordination fragments onto commercial poly-(4-vinylpyridine). Metallopolymers thin films exhibited red, green and blue emission colors defined by Commission Internationale d’Eclairage (CIE) coordinates and medium to excellent photoluminescence (PL) quantum yields (PLQYs) comparable with other highly-performing active materials for Light-Emitting Diodes (LEDs). By grafting a suitable mix of the three different coordination pendants, an efficient single-component white emissive metallopolymer with CIE (0.30, 0.31) was prepared. Thanks to the charged moiety, the polymers resulted miscible with an ionic liquid. The addition produced homogeneous polymeric layers with unaltered PL performances, potentially employable in Light-emitting Electrochemical Cells (LECs).

## 1. Introduction

A light-emitting diode (LED) is a semiconductor light source emitting light undercurrent flow. The color of the light is determined by the band gap of the semiconductor. In particular, white light emission has attracted increasing attention, due to the potential for application in lighting devices and displays. The most common way to realize white-light-emitting devices is based on the three-color-mixing method. Red, green and blue (RGB) light-mixing is obtained by a suitable mix of fluorophores. RGB devices are obtained by a multilayer system with sequential deposition of red, green, and blue emissive species, mixed in a physical blend. In the white organic light-emitting devices (WOLEDs) photoluminescent (PL) organic π-conjugated systems play the leading role. On the other hand, the use of transition metal complexes as emitters in Light-Emitting Diodes (LEDs) technology is well known [1,2,3]. Due to their excellent PL properties and vivid colors, the rare earths ions have been the first choice for LEDs and white LEDs (WLEDs) [4,5,6].

More recently, WPLEDs (white polymer LEDs) based on photoactive polymeric layers have joined, and sometimes take place, of the small-molecular based LEDs [7]. In this approach, a polymer blend usually provides the required tuning of white color. Great efforts have been made to realize a simpler and less expensive manufacturing process [8,9]. To achieve these targets, many research groups have concentrated on polymeric active layers of covalently bonded macro-architecture. Dye-doped blends are often employed in WPLEDs production. However, the unique synthetic versatility and processability and the color tuning of the polymers are irreplaceable. To date, the challenge to obtain easy and cost-effective solid-state active materials for WPLEDs is geared to the design of new intrinsically PL active macromolecules [10,11,12,13,14,15], both organic and metallo-based. Polymers containing transition metal complexes (metallopolymers) represent a relevant varied class of materials. Electron rich ligands coordinated to metal centers can enhance optical and electronic properties [16,17,18,19,20,21,22,23,24,25]. Rare earths containing metallopolymers have received significant interest [26,27] for the noteworthy PL performance, but the toxicity and high process and disposal costs represent a limit on the production of LED devices.

In previous contributions, we explored the role of the metal in tunable highly emissive complexes and in the related metallopolymers, outside of the rare earths group [26,27]. The embedding of the coordination fluorogenic cores into polymeric chains can transfer the emission properties of the micro-environment to a macro-system, with the advantage to match the desired PL performance with the processability of the polymers. Group 12 metal ions, with d^10^ cations, keep a special place, due to the versatility in the structural built and the tunability of PL properties [28]. In addition, zinc ion as a biocompatible low-cost metal is one of the most viable alternatives to rare earths. Due to its closed-shell pattern, zinc ion can preserve or enhance PL performances of the chelating ligands, and this causes a growing interest for industrial applications [29,30,31,32,33]. Processable, stable and cheap PL metallopolymers can be obtained by assembly organic moieties and zinc cations.

**Scheme 1:**
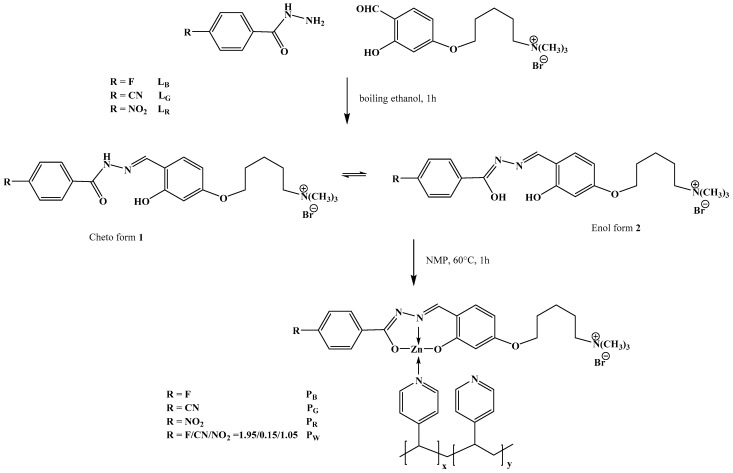
Synthetic route to ligands L_R_, L_G_ and L_B_ and metallopolymers P_R_, P_G_, P_B_ and P_W._

Here we report on the synthesis of three acyl-hydrazone ligands. The compounds bear fluoro, cyano and nitro substituents, and a flexible charged chain guaranteeing solubility in many organic solvents and in water. The hydrazones L_B_, L_G_ and L_R_ (Scheme 1) act as *O*,*N*,*O* ligands toward zinc ion. An easy synthetic route was employed by grafting the coordination moieties to a preformed poly(4-vinylpyridine) (PVPy) to obtain the polymers P_B_, P_G_ and P_R_. By increasing the withdrawing strength of the substituent in the ligand, PL color tuning has been achieved. The solid polymers exhibit respectively blue, green and red luminescence with medium to excellent (82% for the green-emissive polymer) PLQYs. By modulating the contents of various emissive pendants into a single polymer chain, an efficient single-component white emissive material (P_W_) with CIE coordinates (0.30, 0.31) was obtained.

Unlike similar grafted systems [34,35] the charged metallopolymers resulted emissive both in the solid state and in concentered solutions. Moreover, PL performances resulted implemented in color purity and in PLQYs. This feature increases their potential for use in traditional LEDs and even in soft-based light-emitting devices, such as light-emitting electrochemical cells (LECs). LECs are low-cost, easy fabrication lightening devices [16,36,37,38] based on a luminescent polymer mixed with mobile ionic species or intrinsically ionic transition-metal complexes. The introduction of the flexible chain ending with an ionic group [39,40,41] in the ligands made the metallated polymers compatible with an ionic medium. Films of the polymers mixed with the ionic liquid 1-butyl-3-methylimidazolium hexafluorophosphate (BMIM-PF_6_) were obtained, and the basic optical and electronic properties examined.

## 2. Materials and Methods

### 2.1. Materials

All starting products and solvents were commercially available. 5-(4-formyl-3-hydroxyphenoxy)-N,N,N-trimethylpentan-1-aminium bromide was prepared accordingly with a known procedure [42,43] BMIM-PF_6_ and PVPy (Mw = 60,000 and T_g_ = 137 °C) were Sigma-Aldrich products (chemicals, St. Louis, MO, USA). Optical observations were performed by a Zeiss Axioscop polarizing microscope with an FP90 Mettler heating stage (thermogravimetric analysis, PerkinElmer, Inc., Waltham, MA, USA). By using a DSC scanning calorimeter Perkin Elmer Pyris 1 (DSC scanning calorimeter, PerkinElmer, Inc., Waltham, MA, USA) at a scanning rate of 10 °C/min, under nitrogen flow, phase transition temperatures and enthalpies were measured. Thermogravimetric analysis was performed by a Perkin Elmer TGA 4000 (thermogravimetric analysis, PerkinElmer, Inc., Waltham, MA, USA). The zinc content was measured as ZnO residue from TGA analysis. Decomposition temperature was evaluated at 5% weight loss. ^1^H NMR of the ligands and the polymers were recorded in 1,1,2,2-TCE d_2_ using a Bruker Spectrometer operating at 400 MHz. UV-Visible absorption and fluorescence emission spectra were recorded by Jasco F-530 spectrometer (scan rate 200 nm min−1, JASCO Inc., Mary’s Court, Easton, MD 21601, USA) and Jasco FP-750 spectrofluorometer (JASCO Inc., Mary’s Court, Easton, MD, USA) respectively.

### 2.2. Thin Films for Optical Measurements and PLQYs Setup

The thin film sample used for PLQYs were prepared by an SCS P6700 spin-coater apparatus. The *in-situ* reaction of zinc-driven self-assembly afforded thin films of the metallopolymers by spin-coating the NMP reaction mixture onto quartz slides (operating at 600 rpm for 60” in a first step and at 1400 rpm for 60” in a second step) and annealing the films at 150 °C for 10′. The films were washed with isopropyl alcohol twice and dried at 60 °C for 20′.

The samples with BMIM-PF_6_ were obtained from a mixture at 10% wt. of the ionic liquid and the polymer, by spin-coating at 600 rpm for 60” in a first step and at 1400 rpm for 60” in a second step on glass substrates. After annealing at 150 °C for 10′ the films were dried at 60 °C for 20′ in a vacuum.

PLQY of a molecule or material is defined as the ratio of photons absorbed to photons emitted through fluorescence. Photoluminescence quantum efficiency values of thin films of the polymers were recorded on quartz substrates by a Fluorolog 3 (spectrofluorometer, Horiba Jobin Instruments, Kyoto, Japan). Due to the high refractive index of the films, which results in substantial waveguiding of the luminescence, the spectrofluorometer was equipped with an integrating sphere with an optical fibre connection. This overcomes the angular dependence of the emission from film. A measurement is done of the fluorescence emission (*E*_c_) and the scatter (*L*_c_) of the sample and the emission and scatter of a blank (*L*_a_ and *E*_a_). From the two spectral measurements (sample and blank), the PLQY can be calculated from the Equation:

*Φ* = *E*_c_ − (1 − *A*)*E*_b_/*L*_a_∙*A* = *E*_c_ − *E*_a_/*L*_a_ − *L*_c_, (1)

where *E*_b_ is the integrated luminescence from the sample caused by indirect luminescence from the sphere and *A* is the absorbance of the sample at the excitation wavelength.

### 2.3. Synthesis of Ligands L_R_, L_G_ and L_B_

The ligands were synthesized by the same general procedure. As an example, the synthesis of L_B_ is described. To 0.345 g (1.00 mmol) of 5-(4-formyl-3-hydroxyphenoxy)-*N*,*N*,*N*-trimethylpentan-1-aminium bromide dissolved in 15 mL of ethanol 0.146 g (0.95 mmol) of 4-fluoro-benzohydrazide were added. The reaction was kept under stirring at reflux for 30 min. After this time the solution was cooled in an ice bath the precipitation of a whitish crystalline solid ensuing. The solid was recovered by filtration and crystallized from ethanol. Yield: 70%. *M*p = 262 °C. Decomposition temperature: 319 °C. ^1^H NMR (400 MHz, 1,1,2,2-TCE d_2_, 25 °C): *δ* = 1.13 (m, 2 H), 1.90 (m, 2 H), 2.22 (m, 2 H), 3.42 (s, 9H), 3.79 (m, 2H), 4.09 (t, 2H), 6.50 (m, 2 H), 7.27 (m, 3 H), 8.30 (dd, 2H), 9.00 (s, 1 H), 12.02 (s, 1 H), 12.14 (s,1 H) ppm. Elemental analysis calculated (%) for C_22_H_29_N_3_O_3_FBr: C, 54.78; H, 6.06; N, 8.71; found: C, 54.93; H, 6.67; N, 8.70.

L_G_: Yield 77%, *M*p = 290 °C. Decomposition temperature: 302 °C. ^1^H NMR (400 MHz, 1,1,2,2-TCE d_2_, 25 °C): *δ* = 1.19 (m, 2 H), 2.00 (m, 2 H), 2.22 (m, 2 H), 3.44 (s, 9H), 3.80 (m, 2H), 4.08 (t, 2H), 6.47 (s, 1 H), 6.51 (d, 1 H), 7.40 (d, 3 H), 8.22 (m, 2 H), 8.86 (s, 1 H), 11.87 (s, 1 H), 12.00 (s, 1 H) ppm. Elemental analysis calculated (%) for C_23_H_29_N_4_O_3_Br: C, 56.45; H, 5.97; N, 11.45; found: C, 56.43; H, 6.00; N, 11.33.

L_R_: Yield 80%, *M*p = 263 °C. Decomposition temperature: 300 °C. ^1^H NMR (400 MHz, 1,1,2,2-TCE d_2_, 25 °C): *δ* = 1.13 (m, 2 H), 1.90 (m, 2 H), 2.22 (m, 2 H), 3.42 (s, 9H), 3.79 (m, 2H), 4.09 (t, 2H) 6.45 (s, 1 H), 6.49 (m, 1 H), 7.43 (d, 1 H), 8.23 (d, 2 H), 8.33 (m, 2 H), 8.65 (s, 1 H), 11.79 (s, 1 H), 12.02 (s, 1 H) ppm. Elemental analysis calculated (%) for C_22_H_29_N_4_O_5_Br: C, 51.87; H, 5.74; N, 11.00; found: C, 51.43; H, 5.88; N, 11.03.

### 2.4. Synthesis of Metallopolymers P_R_, P_G_, P_B_ and P_W_

The polymers were synthesized by the same general procedure. As an example, the synthesis of P_B_ is described. An amount of 0.900 g (7.43 mmol) dried poly-(4-vinylpyridine) was dissolved in 20 mL of NMP. Ligand L_B_ (0.100 g, 0.207 mmol) was added to the solution under stirring. When all the ligand was dissolved, zinc acetate dihydrate (0.038 g, 0.207 mmol) was added. After 1 h at 60 °C, the solution was poured in 150 mL of methanol. The precipitated polymer was recovered by filtration and purified by dissolution in NMP and reprecipitation in methanol. Yield: 80%. Alternatively, the same solution was employed to produce in situ thin films.

### 2.5. Conductivity Measurements 

In order to determine the electrical conductivity of the films in dark conditions, two silver stripes (300 nm) were thermally evaporated on the top of the investigated films deposited on glass substrates. The conductivity was calculated by using the Ohm law:

(2) $$\sigma =\frac{I\cdot s}{V\cdot w\cdot d},$$

where *I* is the current, *V* the voltage, *s* the distance between two metal stripes, *w* the length of the stripe and *d* the thickness of the film. *I* and *V* were measured by HP 4140B pA meter/DC voltage source in dark condition.

## 3. Results and Discussion

### 3.1. Ligands L_R_, L_G_ and L_B_

Multidentate *O,N* containing ligands as aryl-hydrazones are known for the ability to form stable transition metal complexes with optical and electro-optical properties. The appropriate choice of functional substituents on the ligands and of the metal cation are the factors involved in the tuning of PL performance achievable in the derived coordination complexes [26,44]. The ligands L_R_, L_G_ and L_B_ (in Scheme 1) were obtained by condensation reaction between a 4-substituted-benzohydrazide and 5-(4-formyl-3-hydroxyphenoxy)-N,N,N-trimethylpentan-1-aminium bromide [45]. The compounds undergo keto-enol tautomeric interconversion, where keto-amino tautomer 1 is a more stable form than tautomer 2. In a basic solution, the deprotonated enolate 2 tautomer acts as bi-negative *O*,*N*,*O*-tridentate ligands.

The identification and evaluation of the purity degree were performed by elemental analysis and ^1^H-NMR spectroscopy. Optical observation and differential scanning calorimetry (DSC) was employed to observe crystalline textures and evaluate melting points. Due to the charged chain, the compounds melt after 250 °C with decomposition temperature above 300 °C (see Table 1).

The ligands show low green-yellow emission in diluted ethanol solution (PLQYs 1.5% for L_B_, 0.80% for L_R_ and 2.1% for L_G_) and no relevant solvatochromism depending on the solvent polarity. Contrarily to similar not charged hydrazones [45,46], PLQYs measured on crystalline powder are appreciable for the fluoro and cyano derivatives (3.0% and 5.1% respectively), scarce for the nitro compound (0.7%). The emission maxima in the solid state, recorded in the green-yellow region, resulted slightly red-shifted, depending of the withdrawing strength of the substituent. As expected [45], ligands produce strong emission and an effective color tuning effect only after freezing in the planar rigid conformation, due to zinc coordination. Absorbance and emission maxima recorded in ethanol and on the neat crystalline solids are reported in Table 2.

### 3.2. Polymers P_R_, P_G_, P_B_ and P_W_

We previously proposed a coordination approach based on the use of *O*,*N*,*O* ligands for transition metals as copper, nickel, palladium, cadmium and zinc [25,47,48,49] leading to highly stable complexes. Due to its closed-shell pattern and to the biocompatibility, zinc ion is a good candidate for devices production. This metal resulted able to enhance PL performances of the chelating ligands, determining the color tuning in the final materials. According to previous results [20], when the bi-negative ligand coordinate to zinc (II) ion, the coordination sphere can be completed by neutral pyridine molecules. The ability of pyridine to complete the coordination core of the complex was exploited for preparing metallopolymers by chemical grafting ligand-metal moieties onto commercially available poly(4-vinylpyridine) (PVPy, Mw = 60,000 and *T*_g_ = 137 °C) as summarized in Scheme 1. This approach was demonstrated to produce metallated polymers with nominal concentrations, where the molecular weights of the metallopolymers depend on the grafting percentage and can be adjusted by varying Mw of the preformed chain.

Respect to the common practice of physical doping of a fluorophore in a non-emissive polymeric host matrix, this synthetic strategy represents an easy and cost-effective alternative. The practice experimented before with rare earths metals [44] in our case, adds the advantage of a biocompatible metal and excellent PLQYs at a low grafting percentage. Thanks to the chemical bond between metal centers and polymer chain, undesirable local structuring or phase separation can be avoided. Moreover, a single material is easier to process in the device fabrication.

According to previous studies [35], the best match between good PL performance and processability were obtained in 10 wt.% samples, composition expressed as ligand/(ligand+PVPy)%. This result comes from a particular situation. DFT calculation was previously performed on similar crystalline model complexes. HOMO was found mainly on the molecular plane of the ligand and LUMO on the pyridines that completed the coordination sphere [35]. The environment of the fluorophore units in the polymers resembles a pyridine concentered solution. In the 10% grafted polymers intermolecular interactions among fluorophores are reduced, due to the large number of surrounding pyridine units (where LUMO is located) of the polymeric chain and to the electrostatic repulsions between the cationic chains [50,51,52]. This additional factor justifies PLQYs increased by a factor 10 with respect to strictly similar emissive metallopolymers, without charged chain [35]. Samples with percentage quite higher 10% undergo aggregation caused quenching effect (ACQ) [53,54,55] related to rapid nonradiative decays of the excimers of neighboring fluorophores. Samples with percentages quite lower than 10% show poor emission, due to the scarce number of ligand moieties (where HOMO is located).

**Figure 1:**
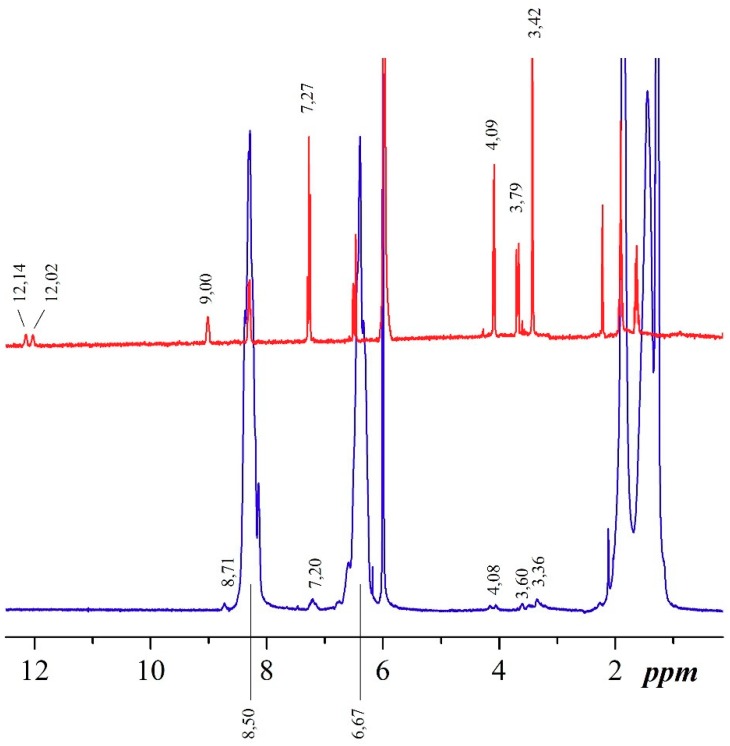
^1^H-NMR spectrum of L_B_ (red curve) and the correspondent metallopolymer P_B_ (blue curve). The diagnostic resonances are evidenced.

The metallopolymers are soluble in common organic solvents and exhibit good viscosity for film deposition (from N-methyl pyrrolidone, NMP, or ortho-dichlorobenzene, o-DCB, concentered solutions). Polymer solutions resulted stable for six months at least. No evidence of crystallinity was detected both in optical observations and in the DSC pattern. Respect to the free PVPy, the glass transition temperature increases from 31 to 51°C as a result of the grafting process (see Table 1). The TGA curves recorded from 50 to 600 °C on the solid samples show no weight loss below 340 °C (Table 1). Zinc content determined as ZnO from the residue at 600 °C (see Experimental Section) is in good agreement with the calculated residue. The ^1^H-NMR spectra generally appear consistent with the expected structure, but the intensity of the NMR signals of the metal-ligand fragments resulted very low with respect to PVPy backbone signals, and not clearly detectable. This behavior is observable in Figure 1, where the ^1^H-NMR spectrum of L_B_ and the correspondent metallopolymer P_B_ are compared.

The spectral pattern of the grafted polymer (blue curve) is consistent with the coordinative bond between the metal-ligand fragment and the PVPy chain. Some of the signals pertaining to the coordination core can be recognized. The resonance ascribable to the tridentate ligand CH=N proton at 8.71 ppm is observed at 9.00 ppm in the free ligand (red curve). Most aromatic resonances are overlapped by the two intense and broad signals centered at 8.50 and 6.67 ppm, due to the polymeric backbone. Only the signal at 7.20 ppm (recorded at 7.27 ppm in the spectrum of the ligand) can be clearly detected. As expected, the signals, due to the NH and OH group (respectively at 12.14 and 12.02 ppm in the cheto form of the ligand) are absent in the polymer spectrum. The signals at 4.08, 3.60 and 3.36 ppm in the blue curve is related to the ones at 4.09, 3.79 and 3.42 ppm (for the ligand), due to OCH_2_, NCH_2_ and N(CH_3_)_3_ groups. Finally, the resonance of a CH_2_ group in the alkoxy chain of **L_B_** is clearly visible at 2.22 in the spectrum of P**_B_**, while the rest is overlapped to the polymeric chain signals, from about 1.05 ppm and 2.10 ppm.

Polymeric thin films were obtained by dissolving the polymer in NMP and spin-coating on quartz slides. A more facile one-step *in-situ* route was also employed. The films produced according to the two methods showed the same properties. As described in the Experimental Section, the reaction mixture of ligand, zinc salt and PVPy can be directly spin-coated on quartz slides. After curing, washing and drying processes, the glassy thin films were employed to explore PL performances in the solid state. The one-step approach not needing a catalyst or initiator is a desirable technique used to depose the emissive layers onto the electrodes in LEDs and other light-emitting devices [56]. According to what observed for analogous systems [35], one or maybe two pyridine molecules could be involved as N donor ligands in penta- or hexacoordinated arrangements. For this reason, no permanent crosslinking is produced in the solid samples. This justifies the high tenacity and adhesion to the quartz substrate and the solubility, at the same time.

**Figure 2:**
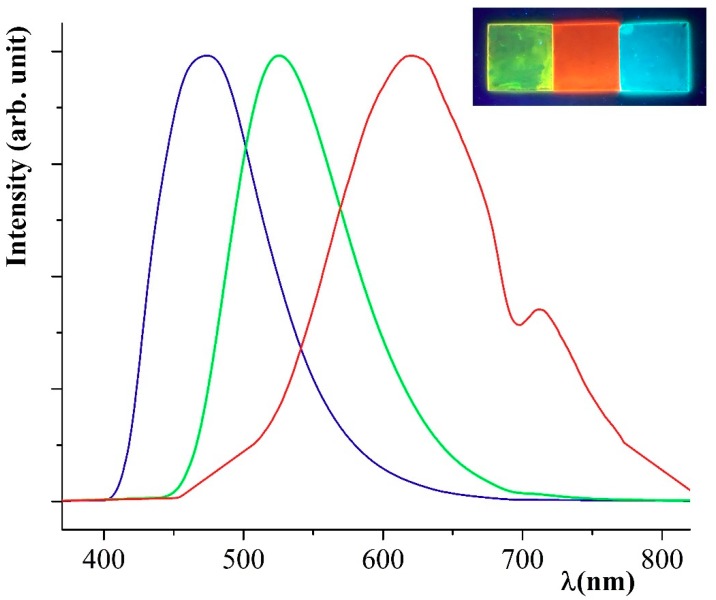
Normalized emission curves recorded on thin film (blue curve) samples of P_B_ (blue curve), P**_G_** (green curve) and P_R_ (red curve). In the inset: The same spin-coated thin films (respectively P_G_, P_R_ and P_B_ from left to right) photographed under the UV lamp at 365 nm.

The polymeric films of P_B_, P_G_ and P_R_ emit respectively in the blue, green and red region of the visible spectrum, as perceptible even to the naked eye (see Figure 2, inset). Commission Internationale de l’Eclairage (CIE) coordinates are (0.17, 0.24), (0.32, 0.56) and (0.54, 0.44) respectively. From medium to large Stokes Shifts (from 88 nm to 185 nm) were recorded, from absorbance (in the visible region) to emission maximum wavelength. This relevant parameter affects the intensity and color purity of the PL emission in LEDs.

PLQYs measured on the blue and on the green luminescent thin films of P_B_ and P_G_ are above 70% (Table 2). As far as we know, 82% measured for P_G_ is unprecedented for a PL metallopolymer. On this base, both polymers can be effective emitters for classical blue and green LEDs. On the other hand, PLQY of P_R_ (25%) is even remarkable because red fluorophores usually provide lower-emission energy, according to the “energy gap low” [57,58]. In all cases, emission resulted implemented both in color purity and in PLQYs respect to the PL metallopolymers we studied in the past [17,25,35,57].

Due to the nature of the charged ligand, the metallopolymers show emission in concentered solutions, while similar not charged systems had worst PL performance [35]. Appreciable PLQYs measured in NMP diluted solutions are from 5.3% (for P_G_) to 4.5% (P_B_) and 1.5% (P_R_). In Table 2 are reported the emission maxima of the same solutions. In a diluted system, the RGB tuning effect is lost, and the emissions are recorded in the lime-yellow region for all samples.

Starting from the information collected on the single metallopolymers, we prepared an efficient single-component white emissive material (P_W_). Since conventional emissive layers in light-emitting devices are composed of three components, morphological stability problems, due to phase separation can occur. In this regards, a single-component emissive polymer incorporating RGB emissive species into a single polymer chain is an intriguing novel approach [59]. In our case, the easiness of the synthesis and of the film deposition makes it the ideal solution.

**Figure 3:**
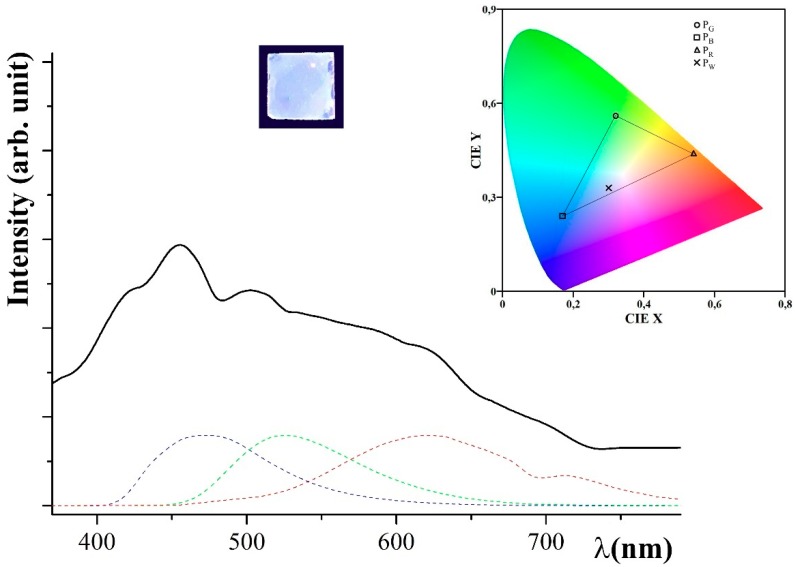
Emission curve recorded on a thin film of P_w_ (black curve). Under P_B_ (blue curve), P_G_ (green curve) and P_R_ (red curve). The same spin-coated thin films photographed under the UV lamp at 255 nm in the inset. CIE 1931 RGB color space for the polymer samples.

A suitable mix of the three different coordination moieties was realized by modulating the contents of the three emissive pendants. The emission in the solid state of the white polymer was realized in the stoichiometric ratio B/G/R = 1.95/0.15/1.05 in moles at 10% total grafting percentage (expressed as ligand/(ligand+PVPy)%.). The red emissive component must be carefully dosed because it causes a decrease in the PL efficiency of the whole system. The spin-coated thin film of P_W_ under UV lamp at 255 nm is pictured in Figure 3 (inset). The fluorescence spectra of the same sample irradiated at 255 nm appear as a single broad band from about 350 to 700 nm. The point in the “RGB triangle” with CIE coordinates of (0.30, 0.31), shown in Figure 3 corresponds to the emission of P_W_, slightly shifted in the blue region, but highly efficient. PLQY measured on the polymer is 37%, to the best of our knowledge, this is an excellent value for a single-component white emissive material in the solid state [60,61,62].

Due to their PL performance, synthetic easiness and processability in the solid state, we demonstrated the potential of the neat polymers for classical LEDs and WPLEDs construction. As potentially active layers of LECs, the first requirement to be met is the compatibility with an ionic medium. This preliminary test was performed by mixing the metallopolymers with the ionic liquid BMIM-PF_6_. Homogeneous thin films of the blends were obtained by spin-coating technique [21,24,63,64,65,66,67,68]. Interestingly, the addition of 10% wt. BMIM-PF_6_ [65] did not make any significant changes in the processability and PL properties (color and PLQY) of the single red, green and blue emissive polymers, and of the white polymer. On the other hand, as a brief glimpse of feasibility, we measured the electrical conductivity in dark conditions of the films with and without the addition of the ionic liquid. As can be seen in Table 3, the conductivity of the red, green, blue and white blend samples deposed on glass substrates increase of a factor from 10^2^ to 10^4^ respect to the neat samples. The conductivity of P_W_ is higher by a factor 10 respect to the green polymer and 100 respect to the blue and red ones. No wonder though values of the electrical conductivity on optimized LEC devices are typically 100 times greater [64,69,70]. The thickness of the films, type and percentage of ionic liquid are relevant parameters that must be optimized. Nevertheless, the collected data can be considered an interesting starting point for the employ of our polymers, also in the fabrication of LECs active layers.

## 4. Conclusions

Three hydrazone tridentate ligands with a different electron-withdrawing substituent and a flexible charged chain were employed as ligand-zinc (II) coordination fragments, grafted onto commercial PVPy. Blue, green and red solid-state emission with CIE coordinates (0.17, 0.24), (0.32, 0.56) and (0.54, 0.44) has been achieved by increasing withdrawing aptitude of the substituent. To the easy synthetic approach, the advantage of a biocompatible metal and good PL performance at low grafting percentage were added. PLQYs measured on in situ spin-coated thin films reach the outstanding 82% PLQY for P**_G_**, which shows great potential as an active layer of green LEDs. By grafting an appropriate choice of different coordination pendants, it was obtained an efficient single-component white emissive metallopolymer based on simultaneous RGB emission, CIE coordinates (0.30, 0.31). With the noteworthy 37% PLQY the white-emissive polymer appears very promising as a single-emissive material for WPLEDs.

The insertion of the charged chain proved to be helpful to make the metallopolymers miscible with an ionic liquid. The potential as LECs active layers were tested on polymer/BMIM-PF_6_ blends, obtaining homogeneous layers with preserved PL performances. Though the thickness of the films and ionic liquid content must be optimized, the conductivity measured on the blends can be considered a promising starting point for actual employ for LECs layers.

## Figures and Tables

**Table 1:** Thermal data of ligands and polymers.

Compound	*M*p (°C) ^(a)^	*T*_g_ (°C) ^(b)^	*T*_d_ (°C) ^(c)^	%ZnO_t_^(d)^	%ZnO_ex_^(e)^
L_B_	262	-	310	-	-
L_G_	290	-	302	-	-
L_R_	263	-	300	-	-
P_B_	-	172	340	1.66	1.70
P_G_	-	188	335	1.64	1.66
P_R_	-	171	330	1.57	1.59
P_W_	-	168	330	1.63	1.60

^(a)^ Melting point. ^(b)^ Glass transition temperature. ^(c)^ Decomposition temperature. ^(d)^ Zinc content calculated as ZnO. ^(e)^ Zn content experimentally determined as ZnO.

**Table 2:** Optical data of ligands and polymers.

Compound	*λ*_abs.sol_(nm)^ (a)^	*λ*_em.sol_(nm) ^(b)^	*λ*_abs.film_(nm) ^(c)^	*λ*_em.film_(nm) ^(d)^	PLQY% ^(e)^
L_B_	329	411(490)	330	501	3.0 ± 0.2
L_G_	339	512	340	521	5.1 ± 0.2
L_R_	346	479	380	570	0.7 ± 0.1
P_B_	344	492	(316)386	474	71 ± 2
P_G_	343	516	(318)430	526	82 ± 2
P_R_	435	502	(329)435	620	25 ± 2
P_W_	354(broad)	400–600(weak)	251(315, 387)	350–700	57 ± 2

^(a)^ Wavelength of UV-Visible absorbance maxima in ethanol solution (ligands) and N-Methyl-2-pyrrolidone (NMP) solution (polymers). ^(b)^ Wavelength of emission maxima in ethanol solution (ligands) and NMP solution (polymers). ^(c)^ Wavelength of UV-Visible absorbance maxima on a thin film obtained from the neat samples. ^(d)^ Wavelength of emission maxima on a thin film obtained from the neat samples. ^(e)^ PL quantum yield measured on thin films.

**Table 3:** Electrical conductivity measured on neat polymeric samples and on 1-butyl-3-methylimidazolium hexafluorophosphate (BMIM-PF_6_) blends.

Compound	BMIM-PF_6_ (% wt.)	Conductivity (μS/m)
P_B_	0	1.9·10^−4^
P_G_	0	4.4·10^−3^
P_R_	0	1.5·10^−4^
P_W_	0	2.3·10^−2^
P_B_	10	5.7·10^−2^
P_G_	10	1.3·10^−1^
P_R_	10	3.4·10^−2^
P_W_	10	1.5
